# Integration of GWAS, pathway and network analyses reveals novel mechanistic insights into the synthesis of milk proteins in dairy cows

**DOI:** 10.1038/s41598-017-18916-4

**Published:** 2018-01-12

**Authors:** Sara Pegolo, Núria Mach, Yuliaxis Ramayo-Caldas, Stefano Schiavon, Giovanni Bittante, Alessio Cecchinato

**Affiliations:** 1 Department of Agronomy, Food, Natural Resources, Animals and Environment (DAFNAE), University of Padua, Viale dell’Università 16, 35020 Legnaro Padua, Italy; 2 UMR 1313, INRA, AgroParisTech, Université Paris-Saclay, 78350 Jouy-en-Josas, France; 3 Animal Breeding and Genetics Program, Institute for Research and Technology in Food and Agriculture (IRTA), Torre Marimon, Caldes de Montbui, 08140 Spain

## Abstract

The quantities and proportions of protein fractions have notable effects on the nutritional and technological value of milk. Although much is known about the effects of genetic variants on milk proteins, the complex relationships among the set of genes and pathways regulating the different protein fractions synthesis and secretion into milk in dairy cows are still not completely understood. We conducted genome-wide association studies (GWAS) for milk nitrogen fractions in a cohort of 1,011 Brown Swiss cows, which uncovered 170 significant single nucleotide polymorphism (SNPs), mostly located on BTA6 and BTA11. Gene-set analysis and the network-based Associated Weight Matrix approach revealed that the milk proteins associated genes were involved in several biological functions, particularly ion and cation transmembrane transporter activity and neuronal and hormone signalling, according to the structure and function of casein micelles. Deeper analysis of the transcription factors and their predicted target genes within the network revealed that *GFI1B*, *ZNF407* and *NR5A1* might act as master regulators of milk protein synthesis and secretion. The information acquired provides novel insight into the regulatory mechanisms controlling milk protein synthesis and secretion in bovine mammary gland and may be useful in breeding programmes aimed at improving milk nutritional and/or technological properties.

## Introduction

Milk is an important source of proteins of high-quality due to their high content of essential amino acids, such as lysine, which is deficient in many human diets^1^, and their well-known physiological effects, such as immunomodulatory and gastrointestinal activities^2^. The main proteins in bovine milk are the four key caseins (CN), namely α_S1_-CN, α_S2_-CN, β-CN and κ-CN, which are organized in micelles and account for about 80% of the total protein content. Casein micelles have a role in concentrating, stabilizing and transporting essential nutrients in milk, mainly Ca^2+^ and proteins, to the offspring^3^. The other protein category is the whey proteins fraction, which consists of mainly β-lactoglobulin (β-LG) and α-lactalbumin (α-LA), immunoglobulins, serum albumin, lactoferrin, lactoperoxidase and a minor component corresponding to glycomacropeptide^3^. This fraction make up approximately 20% of total milk proteins^4^ and it is demonstrated to affect satiety by reducing food intake, stimulating satiating gut hormone production and slowing stomach emptying in humans and animal models (reviewed by Sánchez-Moya *et al*.^5^).

Milk protein content and composition influence milk technological properties (MCP) and are therefore important for the dairy industry, especially in Europe, where the majority of milk produced is transformed into cheese^6^. Milk coagulation, curd structure, curd firmness and cheese yield are directly related to casein content^7^. Additionally, genetic variants of milk protein fractions, and particularly of κ-CN, strongly influence MCPs; κ-CN B milk is indeed characterised by an increased κ-CN content, which favourably affect MCPs^8^. Moreover, milk payment systems in the dairy sectors producing hard cheeses with EU Protected Designation of Origin (PDO) status often include among their payment criteria coagulation and curd firming properties, which are strongly affected by the amounts, proportions and genetic variants of milk protein fractions^8^, as these are related to cheese quality and sensory properties^9,10^. Different milk protein fractions and genetic variants (such as the A1 and A2 variants of β-CN) also seem to affect human health and wellbeing in different ways^11,12^.

In recent decades, there have been extraordinary advances in our knowledge of the physiology and biochemistry of the lactating mammary gland. Despite such efforts, little is as yet known of the genetic regulation of the physiological and cellular mechanisms required for milk protein synthesis and secretion. It is well known that milk protein synthesis in the mammary gland depends on hormonal and developmental cues that modulate the transcriptional and translational regulation of genes through the activity of specific transcription factors, non-coding RNAs and alterations of the chromatin structure in the mammary epithelial cells^13,14^. The interplay between all the aforementioned factors might play a key role in milk protein synthesis, which is crucial during the onset and throughout the lactation in high-producing dairy cattle. Recently, it has also been shown that CN phosphorylation, one of the most important factors controlling the stabilization of calcium phosphate nanoclusters in casein micelles and the internal structure of the casein micelles^15^, is also essential for the protein synthesis machinery in the mammary gland. Differences in the phosphorylation of α_S1_-CN may be of particular interest as it represents 40% of the total CN fraction in bovine milk^16^. The possibility of tailoring milk composition, e.g., to obtain milk with high protein content and/or favourable MCPs, would allow to meet specific demands from the cheese industry and consumers, and therefore represents a highly desirable goal for the dairy industry. Since milk protein composition is less responsive to diet than milk fat content^17^, genomic selection may offer a valid alternative for optimising milk protein nutritional value in relation to human health^7^ while maximizing economic returns for the dairy industry.

There are substantial differences among different bovine breeds in the proportions of milk protein fractions and in the frequencies of protein genotypes^18^. Several studies have investigated the effects of genetic variants of CN and β-LG genes on the milk protein content and cheese-making ability^8,18,19^. However, other *loci* seem to contribute to regulate the proportions and characteristics of milk proteins, suggesting that regulation is shared among different genes^16,20–26^. Deeper knowledge of the set of genes and pathways regulating bovine milk protein synthesis and secretion might, therefore, help to identify their contribution to optimising casein and whey protein contents during lactation. Pathway-based and gene network analyses have been often used as complementary approaches for extracting biological information from genome-wide association analysis studies (GWAS) and for better characterising the genomic structure of complex traits^21,22^.

To date, only one study has explored this type of integrated analysis for milk protein fractions (albeit limited to κ-CN and β-LG and a small cohort of 164 lactating cows), and it suggests that, in addition to the role played by single genes, a complex multi-hormonal system regulates the expression of milk proteins and the interactions between mammary epithelial cells and the components of the extracellular matrix^23^. Nevertheless, no genome-wide association analysis (GWAS) of Brown Swiss populations with the aim of unravelling the genomic architecture controlling milk protein synthesis and secretion has been yet reported. The aims of this study, therefore, were: i) to perform a GWAS analysis to identify genomic regions associated to the proportions of non-protein nitrogen (N) and protein fractions in milk samples from 1,011 Brown Swiss cows; ii) to uncover the biological functions regulating the milk N compound profile through gene-set enrichment analysis; and iii) to use an association weight matrix (AWM) approach^24^ based on SNP co-associations *in silico*, to identify regulatory networks associated with milk protein synthesis, metabolism and secretion in cattle.

## Results

### GWAS analysis

Summary statistics and genomic heritabilities for milk N fractions calculated from a cohort of 1,011 Italian Brown Swiss cows are reported in Table 1. Overall, very high genomic heritabilities were found for the proportions of β-CN (0.833), κ-CN (0.681) and α_S1_-CN (0.661) out of the total nitrogenous compounds. Of the whey proteins, the β-LG proportion also had high heritability (0.558), while the estimates for α-LA were decidedly lower (0.194). Heritabilities of milk non-protein N compounds were moderate (0.363 for minor N compounds, 0.248 for urea).

**Table 1:** Descriptive statistics and genomic heritability $({h}^{2})$ for milk yield and milk nitrogen fractions (n = 1,011).

**Trait** ^1^	Mean	SD	h^2^	#SNP^2^
Milk yield, kg/d	24.26	7.96	0.094	2
True protein N, % total milk N	89.05	2.29	0.402	21
Milk N fractions, % total milk N				
Caseins	77.97	1.25	0.133	4
β-CN	32.14	2.45	0.833	64
κ-CN	9.48	1.48	0.681	74
α_S1_-CN	25.71	1.85	0.661	39
α_S1P_-CN	1.45	0.62	0.171	3
α_S1P_/α_S1_-CN	0.06	0.03	0.183	3
α_S2_-CN	9.19	1.14	0.365	32
Whey proteins	11.08	1.70	0.523	32
β-LG	8.72	1.56	0.558	29
α-LA	2.36	0.51	0.194	7
Other N compounds	10.95	2.28	0.402	21
Minor N compounds	7.94	2.37	0.363	17
MUN	3.01	1.04	0.248	4

^1^True Protein nitrogen (N) and milk N fractions are expressed as percentage of total milk N; α_S2_-CN: α_S2_-casein; α-LA: α-lactalbumin; β-LG: β-lactoglobulin; β-CN: β-casein; κ-CN: κ-casein; α_S1_-CN: α_S1_-casein; α_S1P_-CN/α_S1_-CN: ratio between αS1(phosphorylated)-casein and α_S1_-casein; αS1P-CN: αS1(phosphorylated)-casein; caseins: Σcaseins (β-CN+ κ-CN+ α_S1_-CN+ α_S1P_-CN+ α_S2_-CN+ αS1P/αs1-CN); Whey proteins: Σ whey proteins (α-LA + β-LG). Other N compounds: other N compounds (Σurea + minor N compounds); Minor N compounds: minor N compounds (e.g., small peptides, ammonia, creatine, creatinine, etc.); MUN: milk urea N.

SD: standard deviation; h^2^: genomic heritability.

^2^#SNP: number of significant SNP (5 × ${10}^{-5}$) for each trait.

**Figure 1:**
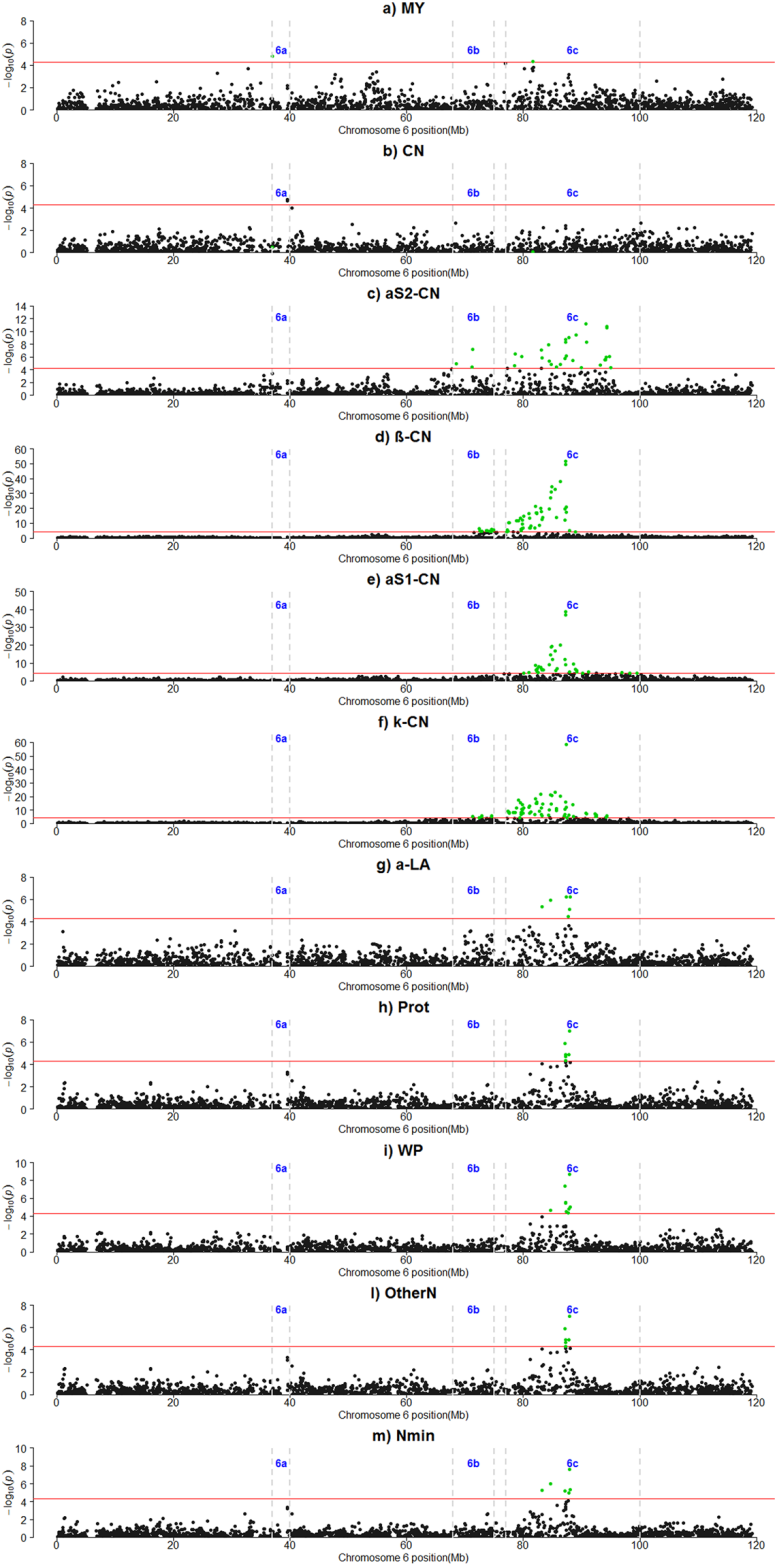
Manhattan plots for the genome-wide association studies on *Bos taurus* autosome 6 (BTA6). (**a**) MY: milk yield; (**b**) CN: Σcaseins (β-CN+ κ-CN+ αS1-CN+ αs1 (phosphorylated)-CN+ αS2-CN+ αS1(phosphorylated)/αS1-CN); (**c**) a_S2_-CN: α_S2_-casein; (**d**) β-CN: β-casein; (**e**) a_S1_-CN: α_S1_-casein; (**f**) k-CN: κ-casein; (**g**) a-LA: α-lactal-bumin; (**h**) Prot: true protein nitrogen (N); (**i**) WP: Σ whey proteins (α-lactalbumin+ β-lactoglobulin); (**l**) OtherN: other N compounds (urea + minor N compounds); (**m**) Nmin: minor N compounds (small peptides, ammonia, creatine, creatinine, etc.). The red horizontal lines indicate a −log10 (*P-*values) of 4.30 (corresponding to *P*-value = 5 × ${10}^{-5}$). 6a: region 6a; 6b: region 6b; 6c: region 6c.

**Figure 2:**
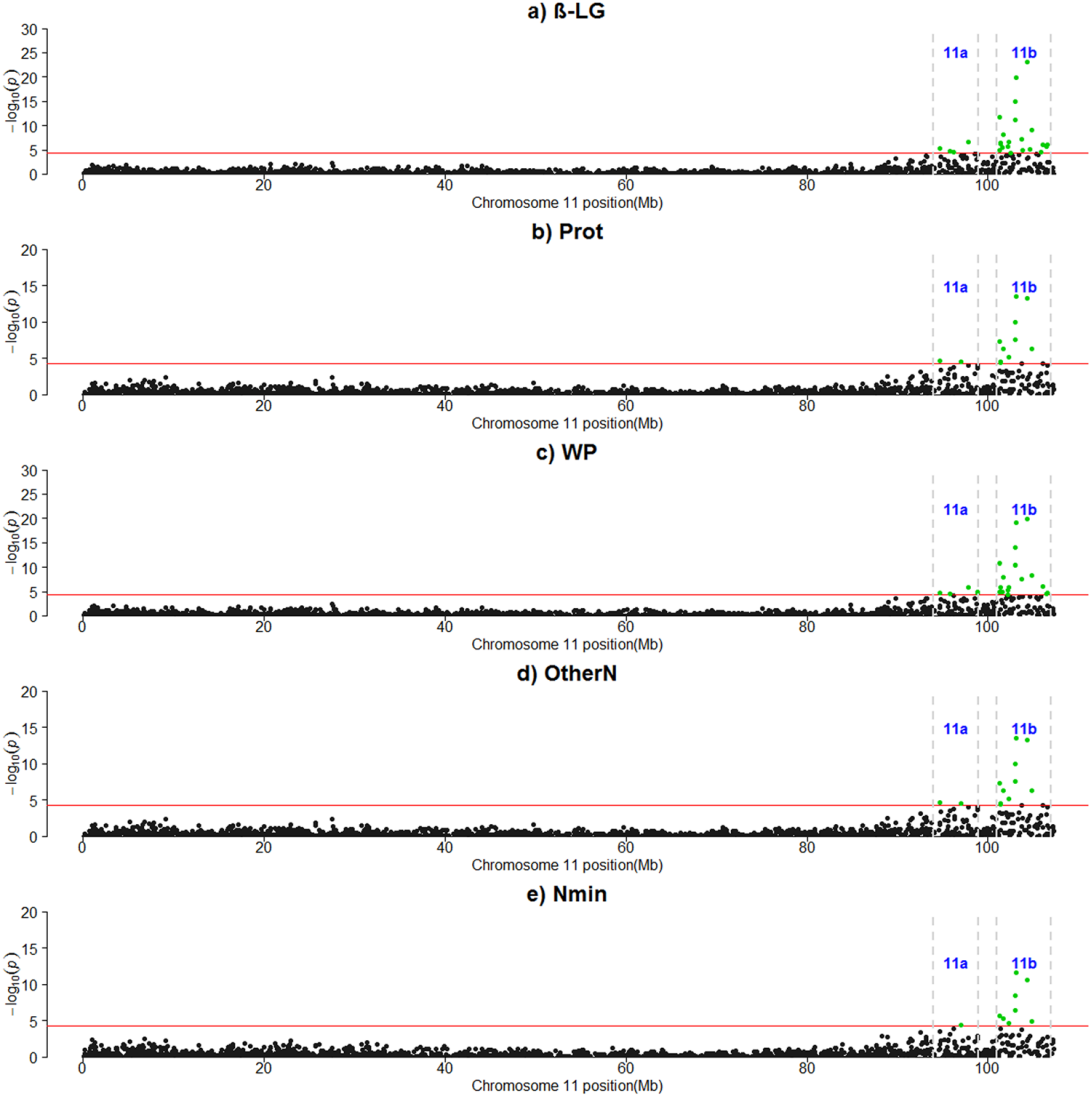
Manhattan plots for the genome-wide association studies on *Bos taurus* autosome 11 (BTA11). (**a**) β-LG: β-lactoglobulin; (**b**) Prot: true protein nitrogen (N); (**c**) WP: whey proteins (β-LG+ α-lactalbumin); (**d**) OtherN: other N compounds (urea + minor N compounds); (**e**) Nmin: minor N compounds (small peptides, ammonia, creatine, creatinine, etc.). The red horizontal lines indicate a −log10 (*P-*values) of 4.30 (corresponding to *P*-value = 5 × ${10}^{-5}$); 11a: region 11a; 11b: region 11b.

Table 2 and Supplementary Table S1 report the results of the GWAS analysis. A total of 170 SNPs were significant, mainly located on two *Bos taurus* autosomes (BTAs), BTA6 and BTA11. Three regions were detected on BTA6, which showed associations with 11 traits (Fig. 1). Region 6a included 3 SNPs (~37.02–39.60) close to the significance threshold associated to the total CN percentage and milk yield (MY). Region 6b (~68.55–74.85 Mbp) corresponded to 17 SNPs associated to α_S2_-CN, β-CN and κ-CN. A total of 103 signals were detected in region 6c (~77.19–99.45) with significant associations with MY, all the CN fractions except for α_S1P_-CN and α_S1P_/α_S1_-CN, the two whey proteins, α-LA and β-LG, and other N compounds except for milk urea (MUN). Very high peaks corresponding to κ-CN, β-CN and α_S1_-CN were detected in this region. In particular, the highest signal corresponded to the marker Hapmap52348-rs29024684 (~87.40 Mbp), which was significantly associated to κ-CN (P = 5.05443E-59). The proportion of additive genetic variance (Va) explained by this SNP was 71.60% (see Supplementary Table S1). Other peaks corresponded to Hapmap28023-BTC-060518 (~87,20 Mbp), which was associated with β-CN (P = 1.72926E-52, Va = 49.67%) and α_S1_-CN (P = 1.2914E-39, Va = 39.56%), and Hapmap24184-BTC-070077 (~87,25 Mbp), which was associated to β-CN (P = 2.60856E-50, Va = 47.55%) (see Supplementary Table S1). Moderate linkage disequilibrium (LD) was observed between Hapmap52348-rs29024684 and Hapmap28023-BTC-060518, and between Hapmap52348-rs29024684 and Hapmap24184-BTC-070077 (r^2^ = 0.35). The markers Hapmap28023-BTC-060518 and Hapmap24184-BTC-070077 were in full LD (r^2^ = 1) (see Supplementary Fig. S1). Two regions were detected on the tail part of BTA11: region 11a, containing 7 significant SNPs (~94.69–98.89 Mbp), and region 11b (~101.27–106.54 Mbp), containing 22 SNPs. Both regions were significantly associated to β-LG, whey proteins, other N compounds and minor N compounds (Table 2) (Fig. 2). The highest signals were detected in region 11b and corresponded to markers ARS-BFGL-NGS-115328 (~103.11 Mbp) associated to β-LG (*P* = 1.12371E-20), and ARS-BFGL-NGS-104610 (~104.29 Mbp) associated to β-LG (*P* = 6.92605E-24) and total WP (*P* = 1.29446E-20). The markers BTA-76907-no-rs and ARS-BFGL-NGS-110734 had undefined positions on the genome and showed highly significant associations with κ-CN (*P* = 2.80E-16) and β-CN (*P* = 6.16E-15) (see Supplementary Table S1).

**Table 2:** Summary results of the genome wide association analysis for milk nitrogen fractions.

**BTA** ^1^	**#SNP**	**Interval, Mbp**	**P-value (range)**	**Top SNP**	**Top SNP location, bp**	**Top SNP MAF**	**Trait** ^2^
1	1	—	2.75E-05	BTB-01778303	151883849	0.02	α_S2_-CN
3	1	—	4.64E-05	ARS-BFGL-NGS-100159	88864456	0.49	α-LA
3	1	—	1.23E-05	ARS-BFGL-NGS-33061	44364191	0.01	CN
4	1	—	3.68E-05	BTB-01672972	21194199	0.01	**Other N**, protein
4	1	—	3.29E-05	BTB-01066453	53857273		**Other N**, protein
4	2	73.60–73.84	(7.34E-06, 2.72E-05)	BTA-71368-no-rs	73837632	0.05	MUN
5	1	—	1.8E-05	Hapmap44167-BTA-95489	82944314	0.07	MUN
6a	3	37.02–39.60	(1.64E-05, 2.23E-05)	Hapmap31921-BTC-033863	37019972	0.05	**MY**, CN
6b	16	68.55–74.85	(5.86E-08, 4.5E-05)	Hapmap29639-BTC-041962	71350048	0.02	**α**_**S2**_**-CN**, β-CN, κ-CN
6c	105	77.19–99.45	(5.05E-59, 4.96E-05)	Hapmap52348-rs29024684	87396306	0.24	**κ-CN**, β-CN, α_S2_-CN, α_S1_-CN, MY, α-LA, Nmin, WP, β-LG, protein, Other N
9	1	—	4.34E-05	BTA-21753-no-rs	36790663	0.01	α_S1_-CN
11a	7	94.69–98.89	(2.36E-07, 3.60E-05)	Hapmap56906-rs29014970	97844929	0.31	**β-LG**, WP, protein, Other N, Nmin
11b	22	101.27–106.54	(6.93E-24, 4.94E-05)	ARS-BFGL-NGS-104610	104293559	0.45	**β-LG**, WP, Other N, protein, Nmin
13	1	—	2.9E-05	ARS-BFGL-NGS-108308	28999095	0.23	MUN
14	1	—	2.16E-05	BTA-02620-rs29010169	45601728	0.01	**α**_**S1P**_**/α**_**S1**_**-CN**, α_S1P_-CN
20	1	—	1.27E-05	ARS-BFGL-NGS-102102	10233876	0.37	**α**_**S1P**_**-CN**, α_S1P_/α_S1_-CN
20	1	—	6.37E-06	Hapmap51592-BTA-41521	46709345	0.37	**α**_**S1P**_**/α**_**S1**_**-CN**, α_S1P_-CN
20	1	—	5.85E-06	BTB-01648552	58264762	0.42	**Protein**, Nmin, Other N
24	1	—	4.22E-05	ARS-BFGL-BAC-42839	4118163	0.11	Nmin
25	1	—	5.19E-06	Hapmap31994-BTC-065943	5385729	0.14	CN

#SNP = number of the single nucleotide polymorphisms significantly associated to the trait; Interval: The region on the chromosome spanned among the significant SNP(s) (in Mb); *P*-value (range) = The *P*-value of the highest significant SNP adjusted for genomic control and the range of the *P*-values when multiple SNP were significantly associated to one trait; Top SNP location (bp) = position of the highest significant SNP on the chromosome in base pairs on UMD3.1 (http://www.ensembl.org/index.html); Top SNP MAF = minor allele frequency of the top SNP.

^2^True Protein nitrogen (N) and milk N fractions are expressed as percentage of total milk N; α_S2_-CN: α_S2_-casein; α-LA: α-lactalbumin; Other N: other N compounds (urea + minor nitrogen compounds); MY: milk yield; β-LG: β-lactoglobulin; β-CN: β-casein; κ-CN: κ-casein; αS1-CN: αS1-casein; Nmin: minor N compounds (e.g., small peptides, ammonia, creatine, creatinine, etc.); α_S1P_/α_S1_-CN: ratio between α_S1_(phosphorylated)-casein and α_S1_-casein; α_S1P_-CN: α_S1_(phosphorylated)- casein; CN: casein, Σcaseins (β-CN+ κ-CN+ α_S1_-CN+ α_S1P_-CN+ α_S2_-CN+ α_S1P_/α_S1_-CN); WP: whey proteins, Σ whey proteins (α-LA + β-LG); MUN: milk urea N.

The trait with the highest *P*-value in each genomic region is bolded.

Adjusting for the effect of the highest signals for κ-CN and β-LG altered the SNPs with the most significant associations (see Supplementary Table S1). The genetic variance explained by the SNPs for the κ-CN proportion decreased dramatically (0.124 *vs* 1.138; −89.1%), as did heritability (0.325 *vs* 0.681; −73.3%). Significant decreases were also observed for the proportions of β-CN (−43.9% genetic variance, −23.5% heritability) and of β-LG, although to a lesser extent (−23.4% genetic variance, −11.0% heritability) (see Supplementary Table S1).

### Pathway analysis

**Figure 3:**
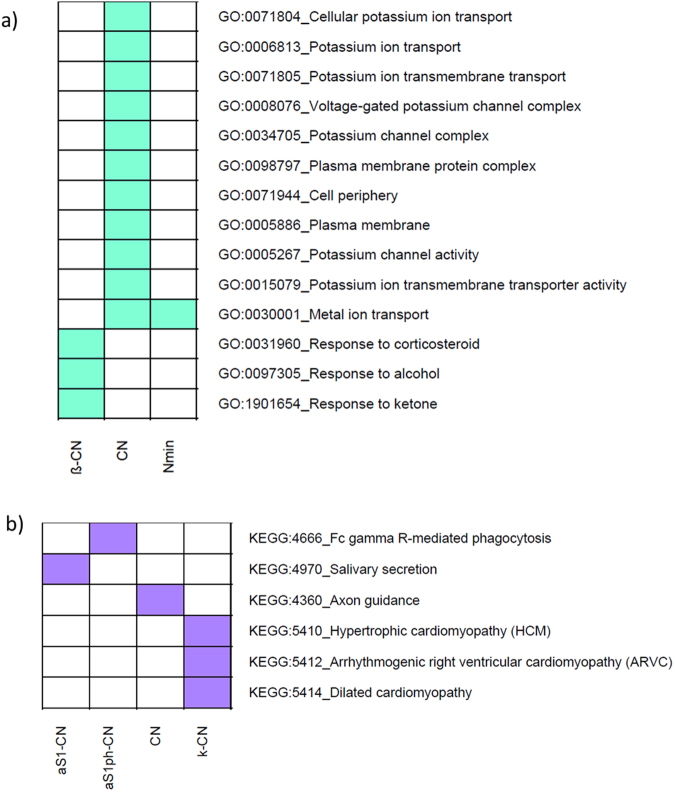
Distribution of the significantly enriched terms/pathways using genes associated to the milk nitrogen fractions. The SNP (*P* < 0.05) were assigned to genes if they were located within the gene or in a flanking region of 15 kb up- and downstream of the gene using the biomaRt R package. For mapping, the Ensembl *Bos taurus* UMD3.1 assembly was used as reference. Gene-set enrichment analysis was carried out using the goseq R package. Only the traits showing significantly enriched terms are reported (*q* < 0.05). (**a**) GO terms; (**b**) KEGG-pathways. β-CN: β-casein; CN: Σcaseins (β-CN+ κ-CN+ α_S1_-CN+ α_S1_phosphorylated-CN+ α_S2_-CN+ α_S1_ (phosphorylated)/α_S1_-CN); Nmin: minor nitrogen compounds; a_S1_-CN: α_S1_-casein; a_S1P_-CN: α_S1_(phosphorylated)-casein; κ-CN: κ-casein.

Of the total 37,568 SNPs used in this study, 17,006 were located in the 15 kb flanking region of the annotated genes. These were assigned to 13,269 genes on the basis of the UMD3.1 bovine genome sequence assembly. On average, a total of 600 genes showed significant associations (*P* < 0.05) with MY or milk N fractions. To gain a better understanding of the functional implications of these 600 significant genes, we performed pathway analyses in order to identify over-represented biological processes. On the one hand, the total CN percentage was significantly enriched by K+ transport pathways, including 7 over-represented gene ontology (GO) categories, e.g., K+ ion transmembrane transport (*q* = 0.00015), voltage-gated K+ channel complex (*q* = 6.07E-06) and K+ channel activity (*q* = 1.16E-05; Fig. 3a). The plasma membrane, plasma membrane protein complex and cell-periphery cellular components were also significantly enriched for CN (*q* = 0.00011, q = 1.33E-05 and q = 8.94E-05, respectively; Fig. 3a). On the other hand, over-represented pathways for β-CN included cellular responses to stimuli, e.g., alcohol (*q* = 2.89E-06), corticosteroid hormones (*q* = 2.30E-05) and ketone bodies (*q* = 4.54E-05; Fig. 3a). Minor N compounds (N min) were significantly associated with the metal ion transport pathways (*q* = 1.04E-05) (Fig. 3a). The full list of significantly enriched pathways (*q* < 0.05) is given in Supplementary Table S2).

Complementary, the most significant over-represented KEGG pathways for κ-CN included genes involved with Ca^2+^ homeostasis, Ca^2+^ cycling and elevation in intracellular Ca^2+^, as well as hypertrophic cardiomyopathy (HCM) processes (*q* = 7.22E-06), arrhythmogenic right ventricular cardiomyopathy (ARVC) (*q* = 2.73E-05) and dilated cardiomyopathy (DCM) (*q* = 8.63E-05; Fig. 3b). Axon guidance was enriched for total CN (*q* = 3.92E-07) while salivary secretion was associated with α_S1_-CN (*q* = 5.20E-05). The Fc γ R-mediated phagocytosis displayed an association with α_S1P_-CN (*q* = 8.86E-05) (Fig. 3b and Supplementary Table S2).

### Gene network analyses

A total of 15,277 annotated SNPs were used for the AWM construction and the SNP co-association analyses. The AWM matrix was then built using a total of 15 phenotypes and the 1,917 SNPs that were significantly associated with at least one of these phenotypes (selected after applying the filtering steps described in the Material and Methods section). These SNPs corresponded to 1,917 unique genes. The SNPs selected by the AWM method explained 72% of the phenotypic variance for κ-CN, which was significantly larger (*P* < 0.001) than the average variance (46%) explained by the same number of randomly selected SNPs (10,000 replicates). Hierarchical clustering of traits was firstly performed to describe the set of phenotypes that inevitable were correlated between them. In fact, milk N fractions profiles were clustered in three different groups: the first comprised the minor N compounds, the second comprised the whey proteins, total CN and the α_S_-CN fraction, while the third included β-CN, κ-CN, urea, α_S1P_-CN and the α_S1P_/α_S1_-CN ratio (Supplementary Fig. S2). Then, operating on the rows of the AWM matrix, the correlations between all pair-wise genes were used to predict gene interactions and generate a regulatory network for the milk N fractions, where the nodes are genes and the edges represent significant interactions between nodes. The PCIT algorithm identified a total of 235,764 edges connecting the 1,917 nodes. After filtering for sparse correlations values ≥ |0.80|, we obtained a regulatory network with 101,284 edges and 1,904 nodes. The analysis of the network topological parameters, e.g., closeness centrality and betweenness centrality, revealed that the genes related to ion transport pathway (e.g., *ITPR2*, *IQGAP1*, *TP53RK* and *LACE1*), protein metabolism (e.g., *METAP1* and *PRC1*) and axon guidance (e.g., *NTNG1* and *ROBO3*) might have an important influence on the regulatory network. Ranking the nodes according to their degree (number of significant interactions), we found *BPIFB1* and *FAM169A* at the top of the list with 481 and 477 edges, respectively (Supplementary Table S3). Analysis with the LASAGNA tool, which predicts the transcription factors (TF) binding sites in the genes’ promoter regions, showed that the promoter of *BPIFB1* and *FAM169A* contained binding sites for several TFs involved in regulating milk protein synthesis, such as *GR*, *ER*, *STAT5A*, *C/EBP* and *YY1* (Supplementary Table S4). Additionally, we detected other highly-connected nodes within our regulatory network, including the K+ channel *KCNK9* (with 455 edges), transporters such as *CRABP1* (450 edges) and *SLC4A7* (420 edges), and the phosphatase *PLPP7* (located 2 Mb from *PAEP*; 418 edges) (Supplementary Table S3).

**Figure 4:**
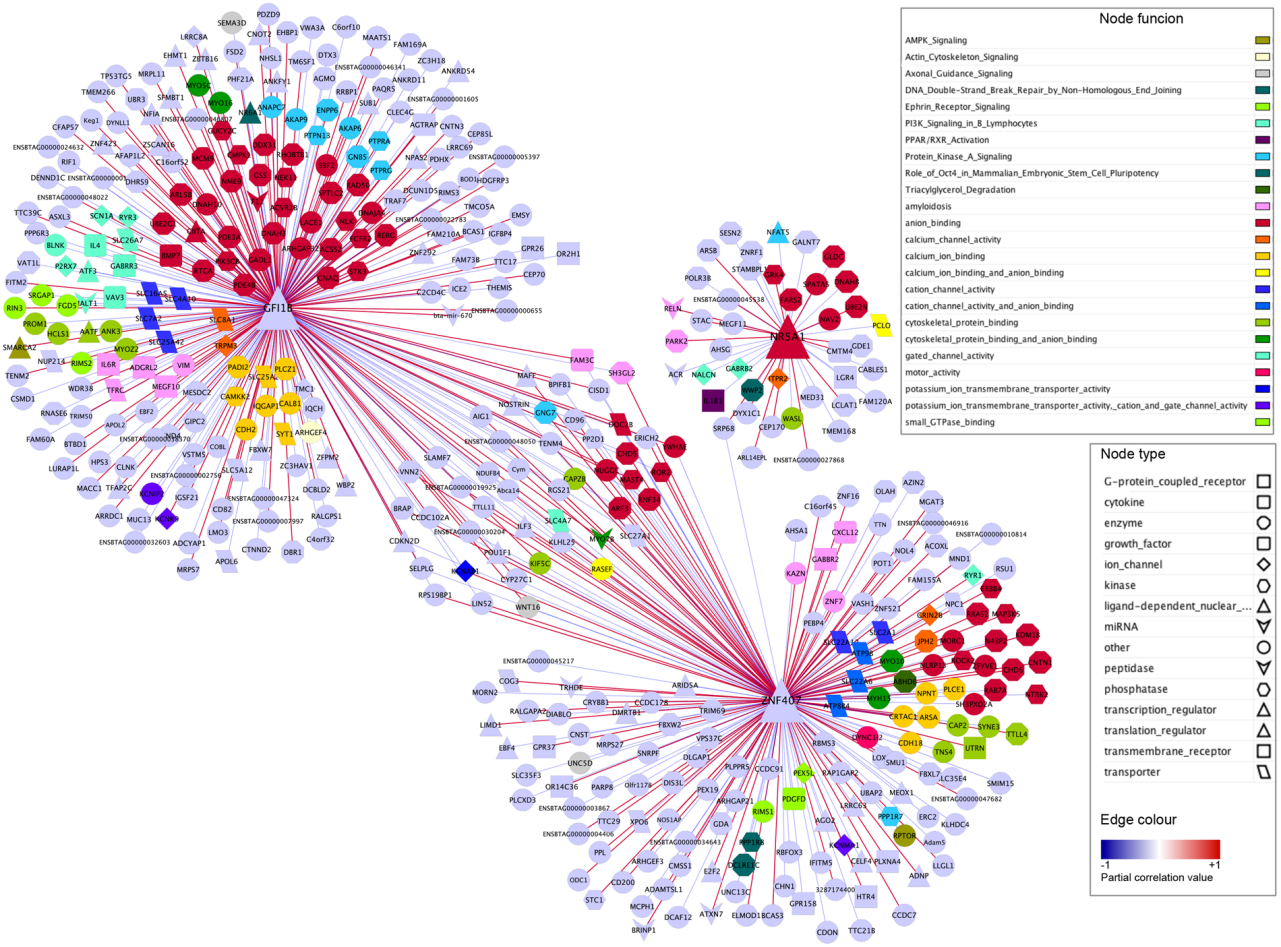
Activators and repressors of the regulatory network of genes associated with the bovine milk κ-casein content. This network contained 452 nodes and 498 edges. In the network, each node represents a gene, whereas every edge connecting two nodes represents a significant interaction (correlation value ≥ |0.80|). In the network, the best trio of transcription factors is showed: *GFI1B, NR5A1* and *ZNF407*. Together they control 2.5% of the regulatory network. The nodes shape indicates whether the node is a transcription factor (triangles), a miRNA (hexagon), a metabolite (round rectangle), a membrane receptor (rectangle), a transporter (parallelogram), or other type of genes (ellipses). The node colour represents the biological function of the gene according to Ingenuity Pathway Analysis (IPA) annotation. The edge colour intensity indicates the level of the association: red = positive correlation - and blue = negative correlation between two nodes.

**Figure 5:**
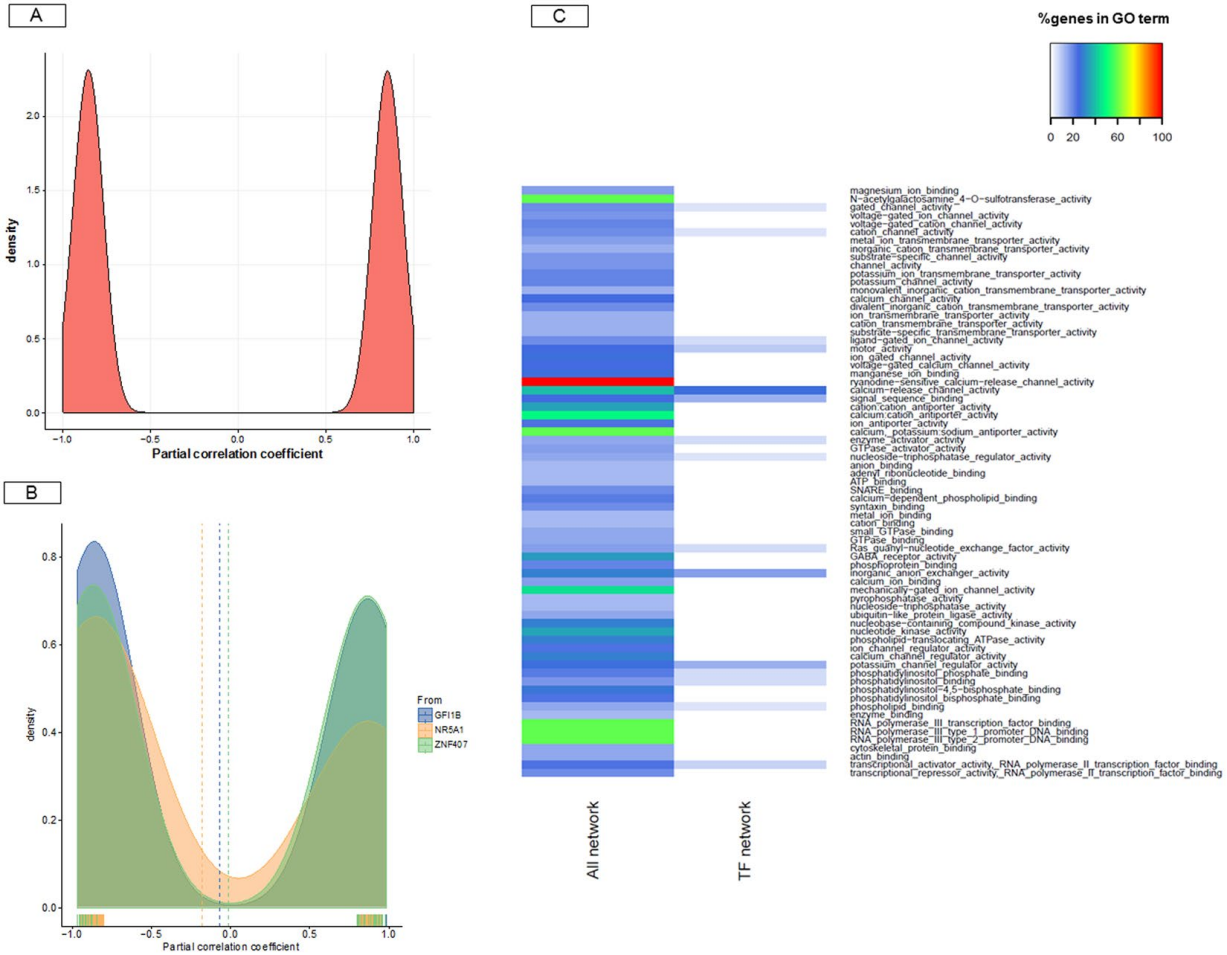
Molecular functions commonly enriched in the full- and the transcription factor-network. Partial correlation values and GO terms displayed between the two types of networks. (**A**) Density plot representation of the partial correlation values in the regulatory network of genes significantly associated with κ-casein profiles in bovine milk; (**B**) Density plot representation of the partial correlation values in the regulatory network based on the three transcription factors. (**C**) The GO term identified with the ClueGO tool were summarized for the two types of network and displayed using a heatmap. The heatmap to show those GO terms that were statistically significant after Benjamini-Hochberg correction (FDR <0.05).

The TFs act in a regulatory network and can drive or repress the expression of different genes in a feed-forward and feedback manner. Accordingly, a second network was generated to explore the main putative regulatory TFs in our regulatory network and the connectivity between them. We identified *GFI1B*, *NR5A1* and *ZNF407* as the “best” trio of TFs within our regulatory network. Altogether, they potentially regulated the transcription of 452 genes (about 24% of genes in the AWM matrix filtered for correlations ≥|0.80|; Fig. 4). Figure 5A,B show the distribution of the partial correlation coefficients in the full and TF networks. More sophisticated regulation patterns between the TFs and their target genes were provided by the LASAGNA promotor analyser. For instance, the promoters of *GFI1B* and *NR5A1* were discovered to contain putative binding sites for the TFs that are known to regulate milk protein synthesis (e.g. *STAT5A*, *C/EBPbeta*, *YY1*, *NFκB*, *NF-1* and *CREB*; Supplementary Table S4; Fig. 4). Differences between the correlation values of the full regulatory network and the TFs network were apparent. The absolute correlation values of the full regulatory network ranged from 0.80 to 1.00, with a mean of 0.86, whereas the absolute correlation values of the TF network ranged from 0.80 to 0.99, with a mean of 0.86. Moreover, while *NR5A1* repressed most of its target genes (63%), the proportion of repressed and induced target genes were similar for *GFI1B* and *ZNF407* (Fig. 5).

To identify the most important cellular activities controlled by the regulatory network and the TFs network, we analysed over-represented GO biological process terms using ClueGO. The full list of enriched pathways and ontologies is reported in Supplementary Table S5. Most of the molecular functions that were commonly enriched in both the full and TF networks were related to ion and cation transmembrane transporter activity and phosphatidylinositol signalling (Fig. 5C). The two networks also shared a considerable number of pathways and biological processes related to neuronal and hormone (e.g. glucocorticoids and insulin) signalling, reproduction, nitrogenous compound metabolism and molecular transport (Supplementary Table S5). Several functions related to the Golgi apparatus were also enriched in both networks such as Golgi vesicle transport, regulation of Golgi organization, intra-Golgi vesicle-mediated transport and post-Golgi vesicle-mediated transport (Supplementary Table S5). In addition, processes and components belonging to the extracellular matrix (ECM), such as the proteinaceous extracellular matrix (*q* = 0.00418), and cell proliferation, e.g., epithelial cell proliferation (q = 0.03981), were significantly overrepresented in the full network (Supplementary Table S5). Immune system response was only over-represented in the TF network, e.g., “positive regulation of lymphocyte mediated immunity” (*q* = 0.03696) and “regulation of adaptive immune response based on somatic recombination of immune receptors built from immunoglobulin superfamily domains” (*q* = 0.04188) (Supplementary Table S5).

## Discussion

### GWAS analysis

We carried out GWAS analysis of the bovine milk N profile, including the main CN and whey protein fractions and non-protein N compounds. The genomic heritabilities we found were generally higher than previously found in the literature, which may be partially due to several factors, such as differences in breed, population size, analytical method, statistical model and data measurement unit (e.g., yield *vs* proportion)^20–26^. Heritabilities of single casein fractions such as κ-CN and β-CN were much higher than that of total caseins. This might be due to the fact that single protein fractions (as well as totals) were expressed as percentage of total N and therefore qualitative (and not quantitative) information was provided. Accordingly, proportions of single milk protein fractions do not share the same profile nor necessarily vary conforming to the totals. The same explanation might be applied also to the number of significant SNPs (much lower in the case of total caseins). However, it is worth mentioning that when using a less stringent *P*- value (as in the case of pathway analyses) the situation was reversed, suggesting that in the case of total caseins the significantly associated signals tended to be mostly weak. These findings might provide further indication that selection for individual milk protein fractions might be more effective than selection based on total caseins, especially when setting breeding programmes aimed at improving milk nutritional and/or technological properties.

As expected, our GWAS results confirmed the highest signals to be on BTA6 in the region of the casein cluster and its flanking region (~86.35–87.40 Mb), and on the tail part of BTA11 including the region of the *PAEP* gene (~101.27–106.54), in line with previous results^20–23^. The most significant SNPs for κ-CN (Hapmap52348-rs29024684), β-CN (Hapmap28023-BTC-060518 and Hapmap24184-BTC-070077, in full LD) and β-LG (ARS-BFGL-NGS-104610 and ARS-BFGL-NGS-115328) are located near (less than 1 Mb from) the causal mutations for protein variants^25,27–35^.

Even after adjusting for the effect of the highest significant SNPs, we still detected high signals on BTA6. Apart from Hapmap28023-BTC-060518 and Hapmap24184-BTC-070077, which are in moderate LD with Hapmap52348-rs29024684, we still found peaks in the ranges 82 to 85 Mb and 88 to 94 Mb. The highest signal in the former region corresponded to Hapmap46932-BTA-111719, which was associated to β-CN, α_S1_-CN and α_S2_-CN. This marker was located about 0.2 Mb from *CTSL2* and 0.5 Mb from *IARS. CTSL2* belongs to the cathepsins family, which are endogenous proteases affecting the physicochemical characteristics of fresh milk and the quality of dairy products; an increase in *CTSL2* expression in bovine milk was observed over the course of lactation^28^. *IARS*, on the other hand, encodes for the isoleucyl-tRNA synthetase. Aminoacyl-tRNA synthetases are key enzymes involved in translating the genetic code by attaching the correct amino acid to each tRNA species and hydrolysing an incorrectly attached amino acid in the editing process^29^. Amino acids serve as precursors for protein synthesis but also act as regulators of protein synthesis^30^. Furthermore, isoleucine seemed to act cooperatively with leucine to increase milk protein synthesis^31,32^, which appeared to be controlled (at least partially) by the mTOR pathway^33^. The highest peak in the latter region corresponded to Hapmap43045-BTA-76998, which was associated to α_S2_-CN and mapped in close proximity to several genes involved in immune system response, e.g., 0.2 Mb from *IL8*, 0.1 Mb from *CXCL6* and 64 Kb from *PPBP*. *IL8*, for instance, is a highly polymorphic gene considered to be a mastitis trait^34^ and may also be a quantitative trait locus (QTL) for milk production traits^35,36^. We found highly significant SNPs on BTA11 in the region flanking *PAEP* (102.94–103.05 Mb) and including the QTL for the β-LG percentage deposited in the Cattle QTL Database. The marker ARS-USMARC-Parent-AY851163-rs17871661 (associated to β-LG, whey proteins, other N compounds, protein and N minor compounds) was located within *GFI1B* (intron variant effect), one of the TFs we proposed as master regulators of milk protein synthesis in bovine mammary gland. We also found a high signal located at 104.29 Mb and corresponding to ARS-BFGL-NGS-104610, which was associated to the same phenotypes. Interestingly, this region (104.13–104.31 Mb) is densely packed with genes coding for small nucleolar-RNA and micro-RNA, well-known regulators of gene expression^37,38^.

### Pathway and network analyses

**Figure 6:**
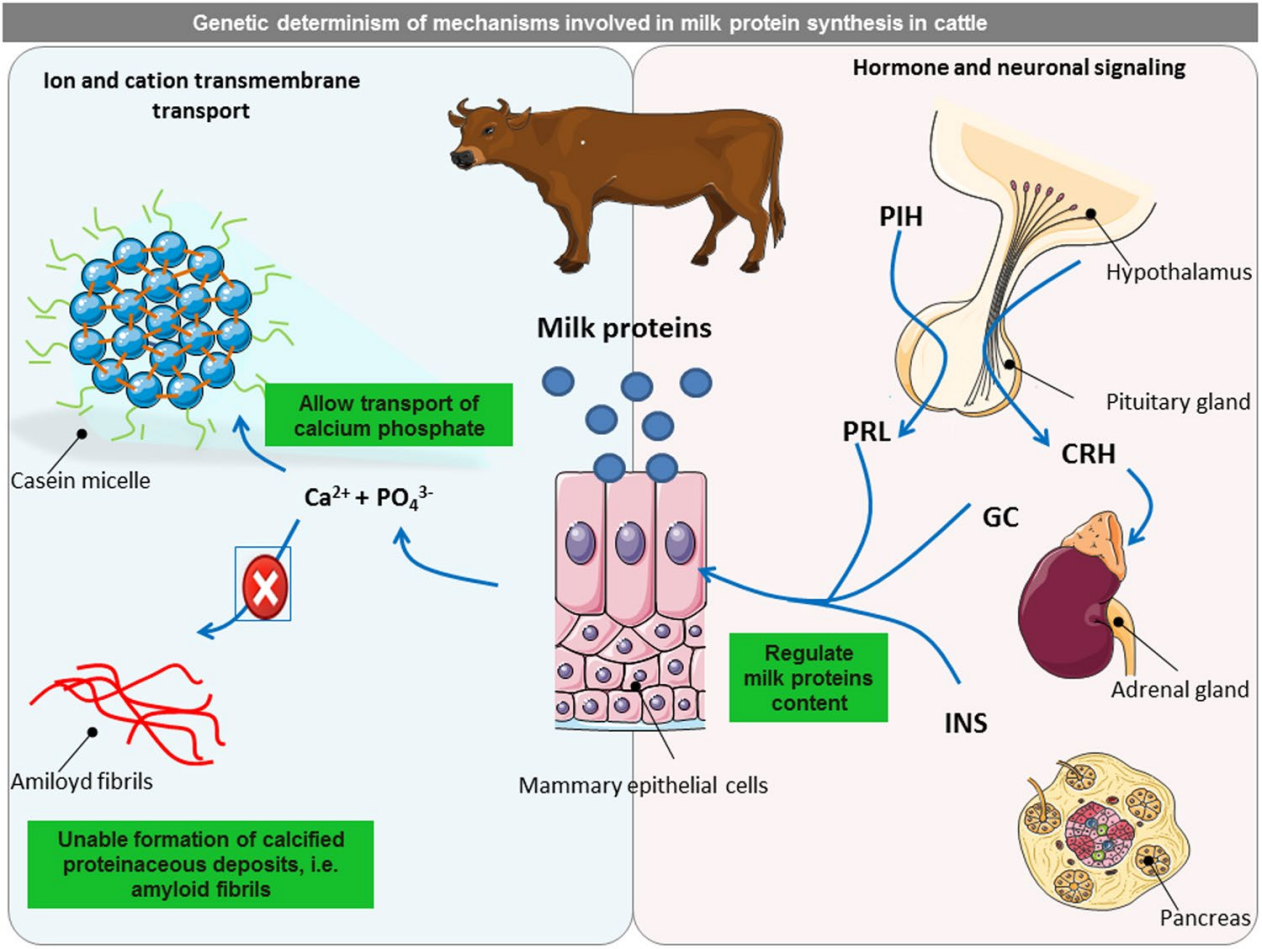
Regulation of milk protein synthesis in bovine mammary gland. The Figure outlines the main significant pathways and cellular functions related to genes associated with milk proteins in bovine mammary gland, including: (i) the regulation of ion and cation transmembrane transport, which is related to the ability of casein micelles to allow transport of calcium phosphate into milk and prevent the formation of calcified, proteinaceous deposits containing amyloid fibrils; and (ii) hormonal and neuronal signaling, particularly through the concerted action of prolactin (PRL), glucocorticoids (GC) and insulin (INS), which are responsible for the regulation of milk protein contents. PIH: prolactin-inhibiting hormone; CRH: corticotropin-releasing hormone; ACTH: adrenocorticotropic hormone. This figure was partly created using images provided by Servier Medical Art (http://www.servier.com/Powerpoint-image-bank/).

Pathway and network analyses derived from GWAS gave additional insights into the complex relationships among genes and the interconnected pathways that are likely to have a role in regulating protein synthesis and secretion in the mammary gland. For instance, we found several pathway associations within our regulatory network, which to the best of our knowledge have not been fully described before, namely: (i) ion and cation transmembrane transport (particularly K+, P, and Ca^2+^); (ii) hormone signalling, (iii) neuronal signalling and (iv) immune system response (Fig. 6; Supplementary Table S5). Additionally, we also identified three TFs, which were likely to be key activators and repressors of a total of 1,904 targets genes within the regulatory network, e.g., *GFI1B*, *ZNF407* and *NR5A1*, which controlled the expression respectively of 260, 197 and 41 genes in the network. Interestingly, many of these pathways derived from GWAS analysis have been also related to milk coagulation properties, curd firmness, cheese yield and curd nutrient recovery^22^, such as calcium and potassium transport, neuronal and hormonal signalling, as well as phosphatidylinositol signalling. These functional findings might confirm the established relationship between milk protein composition and cheese-making traits.

The relationship between CN percentage in milk and the genes involved in the regulation of Ca^2+^ and phosphate transmembrane transport is in line with the structure and the main functions of the casein micelles, which on the one hand act as Ca^2+^-transporting vehicles to supply young mammals with a highly concentrated yet soluble form of calcium phosphate and on the other hand, prevent calcified, proteinaceous deposits containing amyloid fibrils in the mammary gland^39^. Caseins bind Ca^2+^ via highly phosphorylated sequences called phosphate centres present in α_S1_-CN, α_S2_-CN, β-CN^40^. Calcium-dependent CN kinase is responsible for κ-CN phosphorylation before micelle formation and milk secretion^41^. In agreement to our results, the Ca^2+^ ion-binding GO term has been already associated with κ-CN and β-LG in bovine milk^23^. These biologically reasonable associations were further confirmed by the enrichment of several functions related to the Golgi vesicle transport within the full-network and our TFs network. Indeed, the milk proteins newly synthesized in the rough endoplasmic reticulum are transferred to the Golgi apparatus where they are processed for transport to the apical area of the mammary epithelial cells through secretory vesicles^3^. A cardiovascular regulation function through several genes (e.g. *ARVC, HCM*, and *DCM*) has been also associated with κ-CN, suggesting that this protein fraction is involved with the regulation of Ca^2+^ homeostasis. Impaired Ca^2+^ ion regulation (and alteration in insulin signalling) is known to contribute to the pathophysiological effect on cardiomyocyte function^42^. Furthermore, these cardiovascular related pathways also included genes coding for integrins, the major ECM receptors that have been identified as important regulators of mammary epithelial cell growth and differentiation^43^. In relation to these results, pathways pertaining to the extracellular matrix were indeed significantly enriched in our full-regulatory network. Similarly, Gambra *et al*.^23^ reported an association between the extracellular matrix receptors, κ-CN and β-LG concentrations in bovine milk^23^. Besides Ca^2+^ ion, the K+ transport was also enrichment. It is likely that prolactin (PRL), which have a direct role in milk synthesis^33^, activates the extrusion of Na+ and the entry of K+ in mammary cells in both lactating and pre-lactating tissue^44^. Interestingly, a plasmin-induced β-CN breakdown product (fraction 1–28) has been found to act as a potent blocker of K+ channels in bovine mammary epithelia apical membranes^45^.

Our study also showed that milk proteins related genes were associated with the concerted action of hormones such as prolactin, growth hormone, thyroid hormone, corticosteroids, insulin, and growth factors, which are essential for the regulation of milk protein synthesis within the bovine mammary epithelium^33^. Lactogenic hormones enter MECs by diffusion and synergistically bind to milk protein gene promoters. Indeed, the proximal promoters of the β- and κ-CN genes contain so-called lactogenic response elements that harbour binding sites for TFs, which act either as inducers, such as *GR*, *STAT5*, *NF-1* and *C/EBPβ*, or as repressors, such as *YY-1*^46,47^. Remarkably, binding sites for these abovementioned TFs have also been predicted by the LASAGNA tool for the two most important nodes in the full regulatory network, in particular *BPIFB1* and *FAM169A*, and for the two key TFs, *GFI1B* and *NR5A1*. Among the pathways overrepresented in the networks, regulation of insulin secretion and of insulin-like growth factor receptor signalling pathways were included (Supplementary Table S5). A direct effect of insulin on the bovine mammary gland might be mediated by the major milk protein ELF5, which seemed to be regulated by means of phosphoinositide 3-kinase/Akt signalling^48^, which has been identified as playing a central role in lactation^49^. Overrepresentation of phosphatidylinositol signalling (*PI3K*) in the full and TF networks might provide further support for this hypothesis. Both insulin and IGF1 might in turn activate the mTOR signalling pathway, which is crucial for milk protein synthesis^50,51^. Among the enriched genes included in the insulin secretion pathway, *GLUT1 (SLC2A1)* is of particular interest. The large uptake of glucose by the mammary gland during lactation considerably induces the expression of *GLUT1*^33^, which seemed also to be regulated by mTOR^52^. Both *RPTOR* and *GLUT1* were predicted to be targets for *ZNF407* by our TF network.

Additionally, milk proteins associated genes were involved in the activation of neuronal signalling pathways, suggesting an indirect link to the reproduction process and lactation. The overrepresentation of neurotransmitter signalling, such as the cholinergic synapse (enriched in the full network) and axon guidance may be explained by the stimulation of mechanoreceptors in the teat skin, which induces cholinergic nerve impulses with the result that oxytocin is released from the pituitary gland, essential for milk secretion^53^. In fact, the study carried out by Gao *et al*.^54^ provides support to this hypothesis. These authors reported a significant increase in the expression of all CN genes in the bovine mammary gland at the lactation onset^54^, which is reasonably consistent with the need to meet the nutritional requirements of new-born calves. Having established that neuronal signalling appeared to be associated to milk protein components, we also demonstrated that CN could be related to the control of reproduction. The mammary gland is considered as an accessory reproductive organ^55^. This later association may be attributable to several genes involved in the regulation of reproductive process, including *NR5A1*, which plays an important role in various aspects of reproductive development and function^56^ and also regulates gene expression of pituitary gonadotropins, such as the luteinizing hormone (LH) and the follicle-stimulating hormone (FSH)^57^. On the other hand, we found 100 genes in the full network that might be related to amyloidosis disease. Caseins, as other unfolded proteins, tend to form amyloid fibrils and calcified deposits, although to avoid the risk of amyloidosis and calcification, the mammary gland orchestrates different aggregation mechanisms that result in the formation of the casein micelle^58^. Amyloidosis and the production of amyloid proteins have been associated with a variety of so-called protein conformational or protein misfolding diseases (including Alzheimer’s disease, Parkinson’s disease, type-II diabetes)^59^. Caseins have been also found to function as holdase molecular chaperones to prevent the potentially harmful formation of amyloid fibrils^58^, which might explain the enrichment of the signal sequence binding found in our study.

Finally, the enrichment of pathways related to immune response observed for the TF-network might be partly related to the biological role of *GFI1B* which is a transcriptional repressor that plays important roles in the differentiation of several haematopoietic cells^60^. Our findings might be related to the antimicrobial activity of caseins, and specifically of κ-CN^61^; of interest, an overall increase in the immune response and/or in milk antimicrobial activity of the bovine mammary gland has been observed during lactation^62^.

Milk protein composition is subject to the well-known effect of the major genes coding for the various CNs and whey proteins. In our study, the combination of GWAS and pathway and network analyses showed several genes that were coordinated and highly connected between them, making a substantial contribution at different stages of milk protein synthesis. This information advances our understanding of bovine mammary gland functionality and could be helpful to breeding programmes aimed at improving milk quality and/or technological properties. However, altogether, the correlative nature of associations between outcomes from which causality cannot be determined limits the interpretation of our results. Therefore, it is of paramount importance to carry on larger longitudinal studies to explore the causes and the persistency of these interactions. Additionally, the predicted associations need to be biologically validated, e.g., by integrating genomic data with gene expression profiles, by using machine-learning approaches or animal models with knockout genes.

## Methods

### Ethics statement

The cows included in this study belonged to commercial private herds and were not subjected to any invasive procedures. Milk and blood samples were previously collected during the routine milk recording coordinated by technicians working at the Breeder Association of Trento Province (Italy) and therefore authorized by a local authority.

### Phenotypes and genotypes

Individual milk samples were collected from 1,264 Italian Brown Swiss cows from 85 commercial herds located in the Alpine province of Trento (Italy). Details of the animals used in this study and the characteristics of the area are reported in Cipolat-Gotet *et al*.^63^ and Cecchinato *et al*.^64^.

Milk total nitrogen, casein and urea nitrogen (MUN) were measured using a MilkoScan FT6000 (Foss, Hillerød, Denmark). Proportions of the true proteins, e.g., casein fractions (α_S1_-, α_S1P_-, α_S2_-, β- and κ- CN), and whey proteins [β-lactoglobulin (β-LG) and α-lactalbumin (α-LA)] were determined using validated reversed-phase high-performance liquid chromatography (RP-HPLC)^65^. Each fraction was expressed as a percentage of the milk total nitrogen (N) content. These percentages were summed and deducted from the milk total N content to arrive at the proportion of the remaining minor milk N compounds.

The Illumina BovineSNP50 v.2 BeadChip (Illumina Inc., San Diego, CA) was used to genotype 1,152 cows (blood samples were not available for all the phenotyped animals). Quality control excluded markers that do not fulfil the subsequent criteria: call rates >95%, minor allele frequencies >0.5% and no extreme deviation from the Hardy-Weinberg equilibrium (*P* > 0.001, Bonferroni corrected). After filtering, 1,011 cows and 37,568 SNPs were retained for subsequent analyses.

### Genome-wide association study

Genome-wide association analyses (GWAS) were conducted using single-marker regression in the GenABEL R package^66^ and GRAMMAR-GC (Genome-wide Association using Mixed Model and Regression - Genomic Control) with the default function *gamma*^67^. There are 3 steps to the GRAMMAR-GC: firstly, an additive polygenic model with a genomic relationship matrix is fitted; secondly, the residuals obtained from this model are regressed on the SNPs to test for associations; finally, genomic control corrects for conservativeness of the procedure^68^. The polygenic model was:

1 $${\bf{y}}={\bf{X}}\beta +{\bf{a}}+{\bf{e}},$$

where **y** is a vector of the milk N fractions; *β* is a vector with fixed effects of (i) days in milk of the cow (classes of 30 days each), (ii) parity of each cow (classes of 1, 2, 3, ≥4), and (iii) herd-date effect (n = 85); **X** is an incidence matrix connecting each observation to specific levels of the factors in $\beta$. The two random terms in the model were the animal and the residuals, which were assumed to be normally distributed as ${\boldsymbol{a}} \sim N(0,{\bf{G}}{\sigma }_{g}^{2})$ and ${\boldsymbol{e}} \sim N(0,{\bf{I}}{\sigma }_{e}^{2})$, where **G** is the genomic relationship, **I** is the identity matrix, ${\sigma }_{g}^{2}$ is the additive genomic variance and ${\sigma }_{e}^{2}$ the residual variance. The **G** matrix was built in the GenABEL R package, where for a given pair of individuals i and j, the identical by state coefficients (*f*_*i*,*j*_) is calculated as:

2 $${f}_{i,j}=\frac{1}{N}{\sum }_{k}\frac{({x}_{i,k}-{p}_{k})\times ({x}_{j,k}-{p}_{k})}{{p}_{k}\times (1-{p}_{k})}$$

where N is the number of markers used, *x*_*i*,*k*_ is the genotype of the i^th^ individual at the k^th^ SNP (coded as 0, ½ and 1), *p*_*k*_ is the frequency of the “+” allele and k = 1, …, N.

A significance threshold of *P* < 5 × ${10}^{-5}$ was adopted^69^. Manhattan plots were drawn using the *qqman* R package^70^.

SNP variance was calculated as 2pqa^2^, where p is the frequency of one allele, q = 1 − p is the frequency of the second allele and a is the estimated additive genetic effect. Model (1) was also used to estimate the variance components and the genomic heritability of the traits based on the genomic relationship matrix (2). Heritability was estimated as ${h}^{2}=\frac{{\sigma }_{g}^{2}}{{\sigma }_{g}^{2}+{\sigma }_{e}^{2}}$.

To identify secondary association signals, association analysis conditioning on the primary associated SNPs was carried out to test for the presence of other significantly associated SNPs. Therefore, in model (1) we fixed the most significant SNPs on BTA6 and on BTA11 to obtain SNP effect estimates adjusted for the effect of these highly significant SNPs.

The r-squared statistic was chosen to predict the extent of LD. The r^2^ between pairwise SNPs covering the region of CN loci on BTA6 and the region of the β-LG gene (progestagen-associated endometrial protein, *PAEP*) on BTA11 and their respective 1 Mb flanking regions was calculated using the R package LDheatmap^71^.

### Gene-set enrichment and pathway analyses

Pathway analyses were performed as detailed in Dadousis *et al*.^22^ to identify the biological functions regulating the milk N fraction profile. Briefly, the SNPs (nominal *P*-values < 0.05) were assigned to genes if they were located within the gene or within 15 kb of 5′ and 3′ends^72^ using the BiomaRt R package^73,74^ and the Ensembl *Bos taurus* UMD3.1 assembly. Respect to the GWAS analysis, a less stringent significance threshold was adopted since we aimed to detect the effect of less significant SNPs which still contribute to explain phenotypic variability, as associated to genes which are part of biological networks and cellular processes. Combining weaker but related variant signals we can improve the prediction of how these variants might be collectively related to the phenotypes of interest. The Kyoto Encyclopaedia of Genes and Genomes (KEGG)^75^ and the Gene Ontology (GO) databases^76^ were used to define the functional categories associated to the gene sets. To avoid testing broad or narrow functional categories, only GO and KEGG terms with >10 and <1000 genes were inspected. A Fisher’s exact test was applied to each functional category to test for overrepresentation of significant gene sets. A q-value of 0.05 was set as the cut-off for significant enrichments. The gene-set enrichment analysis was performed using the R package goseq^77^.

### SNP co-association and network analyses

The GWAS results were used to build the AWM as described by Fortes *et al*.^24^. The selection criteria favour genes harbouring SNPs with significant associations across related traits. In brief, κ-CN was selected as the key phenotype (due to its greater importance for milk technological properties) and the SNPs that were associated with it (*P* ≤ 0.05) were included in the AWM.

Dependency among phenotypes was explored by estimating the average number of other phenotypes (Ap) that were associated with these SNPs at the same *P* value (*P* ≤ 0.05) (Ap = 3). Then, we selected SNPs that were both close (<10 Kb) to the nearest annotated gene (UMD3.1 assembly) and were associated with any ≥3 other traits (*P* < 0.05). To identify putative regulators, the TFs reported by Vaquerizas^78^ and the microRNA (miRNA) that were mapped to the UMD 3.1 bovine genome assembly (GenBank assembly accession: GCA_000003055.3) were also included in this analysis. To estimate the phenotypic variance explained by the AWM-SNPs, we constructed a first ***G*** matrix based only on the SNPs that were selected for the AWM. The same numbers of randomly selected SNPs were used to build 10,000 **G** matrices (10,000 replicates), to estimate the variance explained by those randomly selected SNPs. The Pearson correlations obtained from pair-wise correlations of AWM columns (standardized SNP effects across traits) were computed and hierarchical clustering of traits was visualised using the hclust function in R^79^. The PCIT algorithm^80^ was used to report significant interactions in the network, which were visualized in Cytoscape^81^. Every node in the network represents a gene, while every edge connecting two nodes represents a significant interaction. In order to include only the high-confidence gene co-associations determined by PCIT, those with correlations ≥|0.80| were retained (n = 1,904 unique genes), on the assumption that these genes have relevant biological significance for the key phenotype from which the AWM-PCIT was constructed. The co-association network was automatically generated using the organic layout algorithm in Cytoscape V2.7 (http://cytoscape.org). Network topological parameters and node centrality values were calculated using the NetworkAnalyzer plugin^82^ to gain insights into the organisation and structure of the complex networks formed by the interacting molecules. In parallel, the list of co-associated genes was fed into the Cytoscape plugin ClueGo^83^ to identify relevant categories of molecular functions, cellular components and biological processes. The ClueGO cut-off for the statistical assessment was FDR < 0.05. In addition, the list of co-associated genes was uploaded to the Ingenuity Pathway Analysis (IPA, version 5.5; Ingenuity Systems, USA) to define information on molecule type (e.g., transcription factor, cytokine, transporter). Genes in the network were coloured according to the biological processes they participate in. Then, a list of TFs (based on Vaquerizas *et al*.^78^) and their target genes, to which they were potentially connected, were identified within our high-confidence gene network (r ≥ |0.80|). An information-lossless approach^84^ was used to identify the optimal subset of TFs spanning the majority of the network topology. The density plots of the genes’ partial-correlation values in the full and the TF network were generated using the R package ggpubr.

Prediction of TF binding sites in the genes’ promoter regions was performed by the LASAGNA-Search 2.0 web tool^85^ using matrices in the TRANSFAC public database and with a significance threshold of *P* = 0.001.

## Electronic supplementary material

Table S1

Table S2

Table S3

Table S4

Table S5

Supplementary information

## References

[1] WHO Technical Report Series PROTEIN AND AMINO ACID REQUIREMENTS IN HUMAN NUTRITION Report of a Joint WHO/FAO/UNU Expert Consultation. At http://apps.who.int/iris/bitstream/10665/43411/1/WHO_TRS_935_eng.pdf. 18330140

[2] Korhonen H, Pihlanto A (2006). Bioactive peptides: Production and functionality. Int. Dairy J..

[3] Rezaei R, Wu Z, Hou Y, Bazer FW, Wu G (2016). Amino acids and mammary gland development: nutritional implications for milk production and neonatal growth. J. Anim. Sci. Biotechnol..

[4] Farrell HM (2004). Nomenclature of the Proteins of Cows’ Milk—Sixth Revision. J. Dairy Sci..

[5] Sánchez-Moya, T. *et al*. *In vitro* modulation of gut microbiota by whey protein to preserve intestinal health. *Food Funct*, **8**, 3053–3063 (2017). 10.1039/c7fo00197e 28636003

[6] European Union. European Commission & European Union Eurostat. *Agriculture, forestry and fishery statistics*. (Publications Office of the European Union, 2016).

[7] Jenkins TC, McGuire MA, Baldwin RL (2006). Major advances in nutrition: impact on milk composition. J. Dairy Sci..

[8] Bittante G, Penasa M, Cecchinato A (2012). Invited review: Genetics and modeling of milk coagulation properties. J. Dairy Sci..

[9] Bittante G (2011). Factors affecting the incidence of first-quality wheels of Trentingrana cheese. J. Dairy Sci..

[10] Bittante G (2011). Monitoring of sensory attributes used in the quality payment system of Trentingrana cheese. J. Dairy Sci..

[11] Bell SJ, Grochoski GT, Clarke AJ (2006). Health Implications of Milk Containing?-Casein with the A^2^ Genetic Variant. Crit. Rev. Food Sci. Nutr..

[12] Graf S, Egert S, Heer M (2011). Effects of whey protein supplements on metabolism. Curr. Opin. Clin. Nutr. Metab. Care.

[13] Rhoads RE, Grudzien-Nogalska E (2007). Translational Regulation of Milk Protein Synthesis at Secretory Activation. J. Mammary Gland Biol. Neoplasia.

[14] Bian Y (2015). Epigenetic Regulation of miR-29s Affects the Lactation Activity of Dairy Cow Mammary Epithelial Cells. J. Cell. Physiol..

[15] Huppertz, T. 1 Proteins-Volume 1A: Basic Aspects in *Advanced Dairy Chemistry Volume 1A* (Eds McSweeney, P. L. H. & Fox, P. F*.)* 135–160 (Springer US, 2013).

[16] Bijl E, van Valenberg H, Huppertz T, van Hooijdonk A, Bovenhuis H (2014). Phosphorylation of αS1-casein is regulated by different genes. J. Dairy Sci..

[17] Lee J, Seo J, Lee SY, Ki KS, Seo S (2014). Meta-analysis of factors affecting milk component yields in dairy cattle. J. Anim. Sci. Technol..

[18] Gustavsson F (2014). Effects of breed and casein genetic variants on protein profile in milk from Swedish Red, Danish Holstein, and Danish Jersey cows. J. Dairy Sci..

[19] Dadousis C (2016). Genome-wide association of coagulation properties, curd firmness modeling, protein percentage, and acidity in milk from Brown Swiss cows. J. Dairy Sci..

[20] Buitenhuis B, Poulsen NA, Gebreyesus G, Larsen LB (2016). Estimation of genetic parameters and detection of chromosomal regions affecting the major milk proteins and their post translational modifications in Danish Holstein and Danish Jersey cattle. BMC Genet..

[21] Peñagaricano F, Weigel KA, Rosa GJM, Khatib H (2013). Inferring Quantitative Trait Pathways Associated with Bull Fertility from a Genome-Wide Association Study. Front. Genet..

[22] Dadousis C (2017). Pathway-based genome-wide association analysis of milk coagulation properties, curd firmness, cheese yield, and curd nutrient recovery in dairy cattle. J. Dairy Sci..

[23] Gambra R (2013). Genomic architecture of bovine κ-casein and β-lactoglobulin. J Dairy Sci..

[24] Fortes MRS (2010). Association weight matrix for the genetic dissection of puberty in beef cattle. Proc. Natl. Acad. Sci. USA.

[25] Schopen GCB (2011). Whole-genome association study for milk protein composition in dairy cattle. J. Dairy Sci..

[26] Bonfatti V, Cecchinato A, Gallo L, Blasco A, Carnier P (2011). Genetic analysis of detailed milk protein composition and coagulation properties in Simmental cattle. J. Dairy Sci..

[27] Huang W (2012). Association between milk protein gene variants and protein composition traits in dairy cattle. J. Dairy Sci..

[28] Wickramasinghe, S., Rincon, G., Islas-Trejo, A. & Medrano, J. F. Transcriptional profiling of bovine milk using RNA sequencing. BMC Genomics **25**,13:45 (2012). 10.1186/1471-2164-13-45 PMC3285075 22276848

[29] Ling J, Söll D (2010). Severe oxidative stress induces protein mistranslation through impairment of an aminoacyl-tRNA synthetase editing site. Proc. Natl. Acad. Sci. USA.

[30] Meijer AJ (2003). Amino acids as regulators and components of nonproteinogenic pathways. J. Nutr..

[31] Appuhamy JA, Knoebel NA, Nayananjalie WA, Escobar J, Hanigan MD (2012). Isoleucine and Leucine Independently Regulate mTOR Signaling and Protein Synthesis in MAC-T Cells and Bovine Mammary Tissue Slices. J. Nutr..

[32] Richert BT, Goodband RD, Tokach MD, Nelssen JL (1997). Increasing valine, isoleucine, and total branched-chain amino acids for lactating sows. J. Anim. Sci..

[33] Bionaz M, Loor JJ (2011). Gene networks driving bovine mammary protein synthesis during the lactation cycle. Bioinform. Biol. Insights.

[34] Ogorevc J, Kunej T, Razpet A, Dovc P (2009). Database of cattle candidate genes and genetic markers for milk production and mastitis. Anim. Genet..

[35] Olsen HG (2002). A Genome Scan for Quantitative Trait Loci Affecting Milk Production in Norwegian Dairy Cattle. J. Dairy Sci..

[36] Boichard D (2003). Detection of genes influencing economic traits in three French dairy cattle breeds. Genet. Sel. Evol..

[37] Shivdasani RA (2006). MicroRNAs: regulators of gene expression and cell differentiation. Blood.

[38] Valadkhan S, Gunawardane LS (2013). Role of small nuclear RNAs in eukaryotic gene expression. Essays Biochem..

[39] Liu J (2016). The Effect of Milk Constituents and Crowding Agents on Amyloid Fibril Formation by κ-Casein. J. Agric. Food Chem..

[40] Holt C, Carver JA (2012). Darwinian transformation of a ‘scarcely nutritious fluid’ into milk. J. Evol. Biol..

[41] Brooks CL, Landt M (1984). Calcium-ion and calmodulin-dependent kappa-casein kinase in rat mammary acini. Biochem. J..

[42] Lebeche D, Davidoff AJ, Hajjar RJ (2008). Interplay between impaired calcium regulation and insulin signaling abnormalities in diabetic cardiomyopathy. Nat. Clin. Pract. Cardiovasc. Med..

[43] Taddei I (2003). Integrins in Mammary Gland Development and Differentiation of Mammary Epithelium. J. Mammary Gland Biol. Neoplasia.

[44] Falconer IR, Rowe JM (1977). Effect of Prolactin on Sodium and Potassium Concentrations in Mammary Alveolar Tissue. Endocrinology.

[45] Silanikove N, Shamay A, Shinder D, Moran A (2000). Stress down regulates milk yield in cows by plasmin induced beta-casein product that blocks K+ channels on the apical membranes. Life Sci..

[46] Rosen JM, Wyszomierski SL, Hadsell D (1999). Regulation of milk protein gene expression. Annu. Rev. Nutr..

[47] Lenasi, T., Kokalj-Vokac, N., Narat, M., Baldi, A. & Dovc, P. Functional study of the equine beta-casein and kappa-casein gene promoters. *J. Dairy Res*. **72****Spec No**, 34–43 (2005). 10.1017/s0022029905001184 16180719

[48] Menzies KK, Lefèvre C, Macmillan KL, Nicholas KR (2009). Insulin regulates milk protein synthesis at multiple levels in the bovine mammary gland. Funct. Integr. Genomics.

[49] Lemay DG, Neville MC, Rudolph MC, Pollard KS, German J (2007). Gene regulatory networks in lactation: identification of global principles using bioinformatics. BMC Syst. Biol..

[50] Castro JJ, Arriola Apelo SI, Appuhamy JADRN, Hanigan MD (2016). Development of a model describing regulation of casein synthesis by the mammalian target of rapamycin (mTOR) signaling pathway in response to insulin, amino acids, and acetate. J. Dairy Sci..

[51] Haar EV, Lee S, Bandhakavi S, Griffin TJ, Kim D-H (2007). Insulin signalling to mTOR mediated by the Akt/PKB substrate PRAS40. Nat. Cell Biol..

[52] Buller CL (2008). A GSK-3/TSC2/mTOR pathway regulates glucose uptake and GLUT1 glucose transporter expression. AJP Cell Physiol..

[53] *Handbook of Milk of**Non-Bovine Mammals*. (Eds Park, Y. W. & Haenlein, G. F. W.) (John Wiley & Sons, 2008).

[54] Gao Y, Lin X, Shi K, Yan Z, Wang Z (2013). Bovine Mammary Gene Expression Profiling during the Onset of Lactation. PLoS One.

[55] Blackburn DG, Hayssen V, Murphy CJ (1989). The origins of lactation and the evolution of milk: a review with new hypotheses. Mamm. Rev..

[56] Richards JS (2002). Novel Signaling Pathways That Control Ovarian Follicular Development, Ovulation, and Luteinization. Recent Prog Horm Res.

[57] Haisenleder DJ, Yasin M, Dalkin AC, Gilrain J, Marshall JC (1996). GnRH regulates steroidogenic factor-1 (SF-1) gene expression in the rat pituitary. Endocrinology.

[58] Holt C, Carver JA, Ecroyd H, Thorn DC (2013). Invited review: Caseins and the casein micelle: Their biological functions, structures, and behavior in foods. J. Dairy Sci..

[59] Ashraf GM (2014). Protein misfolding and aggregation in Alzheimer’s disease and type 2 diabetes mellitus. CNS Neurol. Disord. Drug Targets.

[60] van der Meer LT, Jansen JH, van der Reijden BA (2010). Gfi1 and Gfi1b: key regulators of hematopoiesis. Leukemia.

[61] Meredith-Dennis, L. *et al*. Composition and Variation of Macronutrients, Immune Proteins, and Human Milk Oligosaccharides in Human Milk From Nonprofit and Commercial Milk Banks. *J. Hum. Lact*. 089033441771063 (2017). 10.1177/0890334417710635 28614672

[62] Loor JJ, Moyes KM, Bionaz M (2011). Functional Adaptations of the Transcriptome to Mastitis-Causing Pathogens: The Mammary Gland and Beyond. J. Mammary Gland Biol. Neoplasia.

[63] Cipolat-Gotet C, Cecchinato A, De Marchi M, Bittante G (2013). Factors affecting variation of different measures of cheese yield and milk nutrient recovery from an individual model cheese-manufacturing process. J. Dairy Sci..

[64] Cecchinato A, Albera A, Cipolat-Gotet C, Ferragina A, Bittante G (2015). Genetic parameters of cheese yield and curd nutrient recovery or whey loss traits predicted using Fourier-transform infrared spectroscopy of samples collected during milk recording on Holstein, Brown Swiss, and Simmental dairy cows. J. Dairy Sci..

[65] Bonfatti V, Grigoletto L, Cecchinato A, Gallo L, Carnier P (2008). Validation of a new reversed-phase high-performance liquid chromatography method for separation and quantification of bovine milk protein genetic variants. J. Chromatogr. A.

[66] GenABEL project developers GenABEL: genome-wide SNP association analysis. R package version 1.8–0, https://cran.r-project.org/web/packages/GenABEL/index.html (2013).

[67] Amin N, van Duijn CM, Aulchenko YS (2007). A Genomic Background Based Method for Association Analysis in Related Individuals. PLoS One.

[68] Svishcheva GR, Axenovich TI, Belonogova NM, van Duijn CM, Aulchenko YS (2012). Rapid variance components-based method for whole-genome association analysis. Nat. Genet..

[69] Burton PR (2007). Genome-wide association study of 14,000 cases of seven common diseases and 3,000 shared controls. Nature.

[70] Turner, S. D. qqman: an R package for visualizing GWAS results using Q-Q and manhattan plots, *bioRxiv* (2014).

[71] Shin, J.-H. *et al*. LDheatmap: An R Function for Graphical Display of Pairwise Linkage Disequilibria Between Single Nucleotide Polymorphisms. *J. Stat. Softw*. **016**, (2006).

[72] Pickrell JK (2010). Understanding mechanisms underlying human gene expression variation with RNA sequencing. Nature.

[73] Durinck S (2005). BioMart and Bioconductor: a powerful link between biological databases and microarray data analysis. Bioinformatics.

[74] Durinck S, Spellman PT, Birney E, Huber W (2009). Mapping identifiers for the integration of genomic datasets with the R/Bioconductor package biomaRt. Nat. Protoc..

[75] Ogata H (1999). KEGG: Kyoto Encyclopedia of Genes and Genomes. Nucleic Acids Res..

[76] Ashburner M (2000). Gene ontology: tool for the unification of biology. The Gene Ontology Consortium. Nat. Genet..

[77] Young MD, Wakefield MJ, Smyth GK, Oshlack A (2010). Gene ontology analysis for RNA-seq: accounting for selection bias. Genome Biol..

[78] Vaquerizas JM, Kummerfeld SK, Teichmann SA, Luscombe NM (2009). A census of human transcription factors: function, expression and evolution. Nat. Rev. Genet..

[79] Ihaka R, Gentleman R (1996). R: A Language for Data Analysis and Graphics. J. Comput. Graph. Stat..

[80] Reverter A, Chan EKF (2008). Combining partial correlation and an information theory approach to the reversed engineering of gene co-expression networks. Bioinformatics.

[81] Shannon P (2003). Cytoscape: A Software Environment for Integrated Models of Biomolecular Interaction Networks. Genome Res..

[82] Assenov Y, Ramirez F, Schelhorn S-E, Lengauer T, Albrecht M (2008). Computing topological parameters of biological networks. Bioinformatics.

[83] Bindea G (2009). ClueGO: a Cytoscape plug-in to decipher functionally grouped gene ontology and pathway annotation networks. Bioinformatics.

[84] Reverter A, Fortes MRS (2013). Breeding and Genetics Symposium: building single nucleotide polymorphism-derived gene regulatory networks: Towards functional genomewide association studies. J. Anim. Sci..

[85] Lee C, Huang C-H (2013). LASAGNA-Search: an integrated web tool for transcription factor binding site search and visualization. Biotechniques.

