# Supplementary material for: Integration of GWAS, pathway and network analyses reveals novel mechanistic insights into the synthesis of milk proteins in dairy cows
Source: Sci Rep. 2018 Jan 12;8:566. doi: 10.1038/s41598-017-18916-4 (PMC5766549; doi:10.1038/s41598-017-18916-4)
Supplement: Supplementary file 6 — Supplementary information [file 41598_2017_18916_MOESM6_ESM.docx]

**Integration of GWAS, pathway and network analyses reveals novel mechanistic insights into the synthesis of milk proteins in dairy cows.**

**Sara Pegolo**^1^***, Núria Mach**^2^**, Yuliaxis Ramayo-Caldas**^2,3^**, Stefano Schiavon**^1^, **Giovanni Bittante**^1^ **& Alessio Cecchinato**^1^

^1^ Department of Agronomy, Food, Natural Resources, Animals and Environment (DAFNAE), University of Padua, Viale dell’Università 16, 35020 Legnaro, Padua, Italy

^2^ UMR 1313, INRA, AgroParisTech, Université Paris-Saclay, 78350 Jouy-en-Josas, France

^3^Animal Breeding and Genetics Program, Institute for Research and Technology in Food and Agriculture (IRTA), Torre Marimon, Caldes de Montbui, 08140, Spain

*Corresponding author: [sara.pegolo@unipd.it](mailto:sara.pegolo@unipd.it)


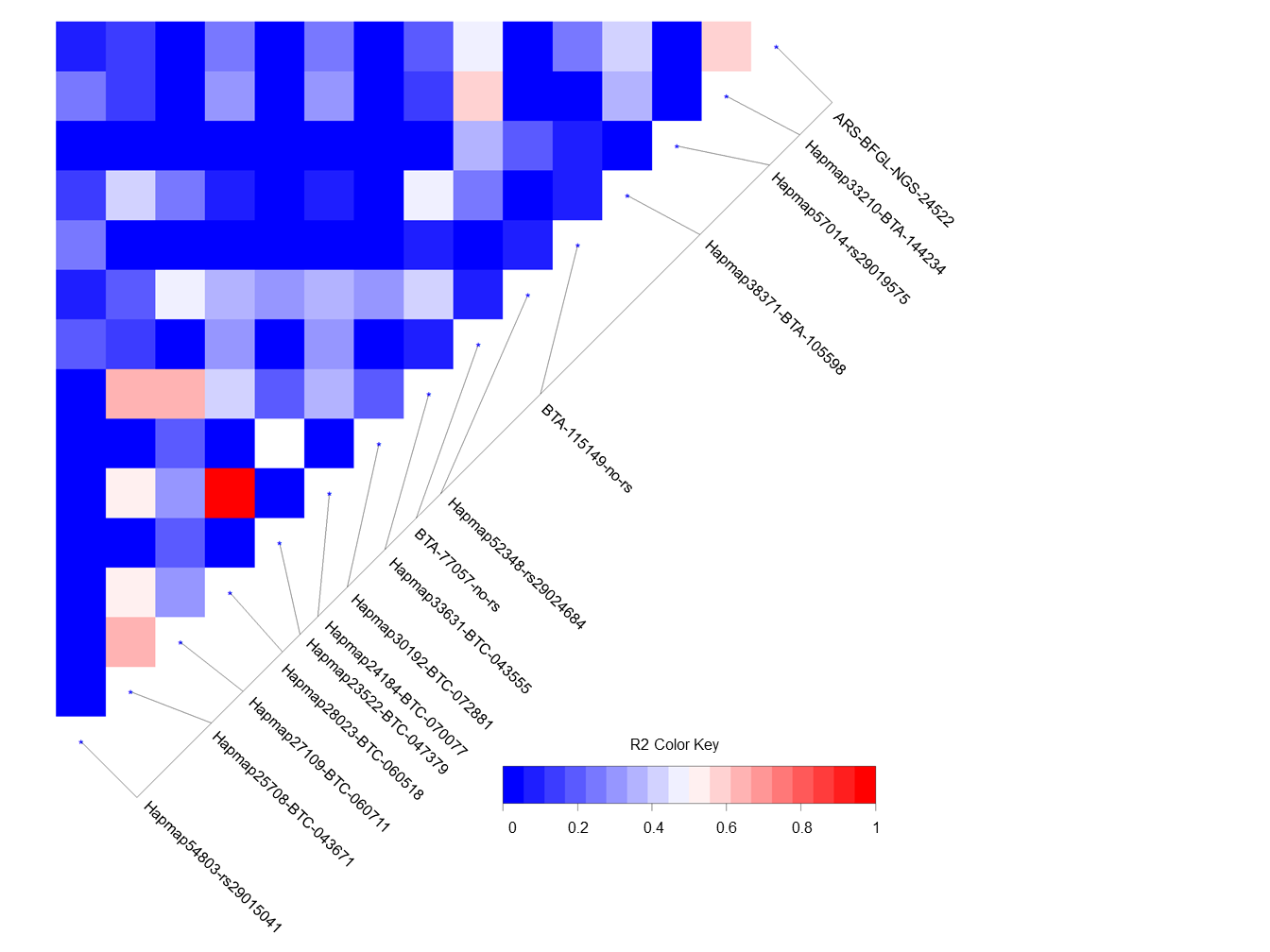


a)

**
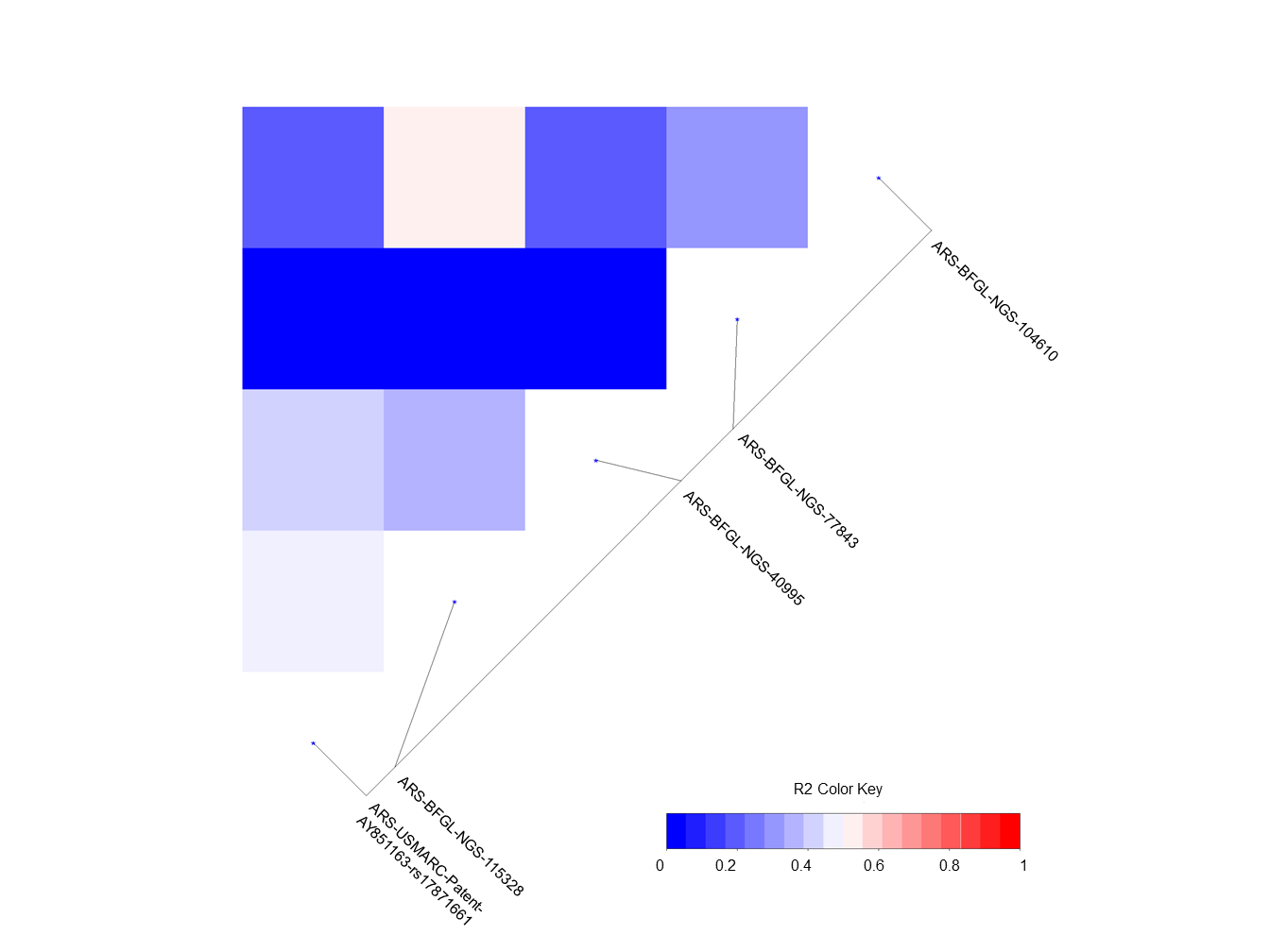
**

b)

**Supplementary Fig. S1. Linkage disequilibrium map for BTA6 and BTA11.** The r2 between pairwise SNPs covering a) the casein locus and 1Mb flanking region and b) the *PAEP* gene and 1Mb flanking region were considered. The R package LDheatmap was used to calculate r2 values.

**Supplementary Fig. S2 .** Hierarchical cluster analysis of the milk nitrogen fractions analyzed in this study.TP: true protein; WP: whey proteins; bLG: β-lactoglobulin; CN: total caseins; aLA: α-lactalbumin; as1CN: αS1-casein; as2CN: αS2-casein; bCN: β-casein; MY: milk yield; MUN: milk urea nitrogen; kCN: κ-casein; as1PCN: αS1(phosphorylated)–casein; as1Ph_as1CN: αS1(phosphorylated)–casein/ αS1-casein.
